# Association of multiparametric MRI quantitative imaging features with prostate cancer gene expression in MRI-targeted prostate biopsies

**DOI:** 10.18632/oncotarget.10523

**Published:** 2016-07-11

**Authors:** Radka Stoyanova, Alan Pollack, Mandeep Takhar, Charles Lynne, Nestor Parra, Lucia L.C. Lam, Mohammed Alshalalfa, Christine Buerki, Rosa Castillo, Merce Jorda, Hussam Al-deen Ashab, Oleksandr N. Kryvenko, Sanoj Punnen, Dipen J. Parekh, Matthew C. Abramowitz, Robert J. Gillies, Elai Davicioni, Nicholas Erho, Adrian Ishkanian

**Affiliations:** ^1^ Department of Radiation Oncology, University of Miami Miller School of Medicine, Miami, FL, USA; ^2^ Reserach and Development, GenomeDx Biosciences, Vancouver, BC, Canada; ^3^ Department of Urology, University of Miami Miller School of Medicine, Miami, FL, USA; ^4^ Department of Radiology, University of Miami Miller School of Medicine, Miami, FL, USA; ^5^ Department of Pathology and Laboratory Medicine, University of Miami Miller School of Medicine, Miami, FL, USA; ^6^ Cancer Imaging and Metabolism, Moffitt Cancer Center, Tampa, FL, USA

**Keywords:** prostate cancer, multiparametric MRI, MRI-targeted biopsies, gene expression, radiogenomics

## Abstract

Standard clinicopathological variables are inadequate for optimal management of prostate cancer patients. While genomic classifiers have improved patient risk classification, the multifocality and heterogeneity of prostate cancer can confound pre-treatment assessment. The objective was to investigate the association of multiparametric (mp)MRI quantitative features with prostate cancer risk gene expression profiles in mpMRI-guided biopsies tissues.

Global gene expression profiles were generated from 17 mpMRI-directed diagnostic prostate biopsies using an Affimetrix platform. Spatially distinct imaging areas (‘habitats’) were identified on MRI/3D-Ultrasound fusion. Radiomic features were extracted from biopsy regions and normal appearing tissues. We correlated 49 radiomic features with three clinically available gene signatures associated with adverse outcome. The signatures contain genes that are over-expressed in aggressive prostate cancers and genes that are under-expressed in aggressive prostate cancers. There were significant correlations between these genes and quantitative imaging features, indicating the presence of prostate cancer prognostic signal in the radiomic features. Strong associations were also found between the radiomic features and significantly expressed genes. Gene ontology analysis identified specific radiomic features associated with immune/inflammatory response, metabolism, cell and biological adhesion. To our knowledge, this is the first study to correlate radiogenomic parameters with prostate cancer in men with MRI-guided biopsy.

## INTRODUCTION

Treatment recommendations for prostate cancer patients are currently based on risk stratification using PSA, Gleason score (GS) and T-category, which typically categorize men as having low, intermediate, and high risk disease [1]. The overtreatment of men with prostate cancer is a well-recognized problem and active surveillance has rapidly become a standard recommendation for many men with low risk disease [2]. Prostate tumor heterogeneity confounds the selection of men for active surveillance or definitive primary treatment because the determinate lesion is missed in approximately 30% of cases. New methods are needed to improve risk stratification and optimize management [3].

Genomic analyses and gene expression signatures, such as Decipher^®^ (GenomeDx, San Diego, California) [4-7], Prolaris^®^ Cell Cycle Progression (CCP) (Myriad Genetics, Salt Lake City, Utah) [8], Genomic Prostate Score^®^ (GPS) (Genomic Health, Redwood City, CA) [9] have the potential to become integral to risk stratification and management. Prostate cancer, however, exhibits spatial heterogeneity that can confound current pre-treatment clinicalopathological and genomic assessment. Multiparametric (mp)MRI, including sequences for anatomical (T2-weighted (T2w)), perfusion (Dynamic contrast enhanced (DCE-)MRI) and diffusion (diffusion weighted imaging (DWI)) is an excellent tool for visualization of prostate structures, distributions of blood flow or diffusion. We propose the characterization of prostate “habitats”, and hence prostate cancer heterogeneity, through identification of distinct imaging characteristics using these sequences [10]. Our approach incorporates “radiomics”, an emerging method for high-throughput extraction of imaging features from diagnostic radiographic series [11]. Quantification and characterization of these features have been found to reflect tumor molecular characteristics (radiogenomics) and, hence, heterogeneity in solid tumors [12].

In this report, we describe for the first time the relationship between quantitative mpMRI and gene expression in prostate cancer samples from patients undergoing mpMRI-directed prostate biopsies. Biopsy targets were selected based on habitats at risk of harboring cancer. We extend the use of the habitat concept to the entire prostate as an important component of tumor microenvironment, including ‘normal’ appearing tissues (NAT) in the peripheral (PZ) and transition (TZ) zones. We demonstrate significant associations between quantitative imaging features and genes associated with adverse outcome, as well as genes associated with specific biological processes from mpMRI-directed biopsy tissue. We show that both tumor and surrounding prostate tissue contribute significantly to radiogenomic features associated with tumor molecular characteristics related to aggressive behavior.

## RESULTS

### Workflow for co-registration of genomic and radiomic data

**Figure 1:**
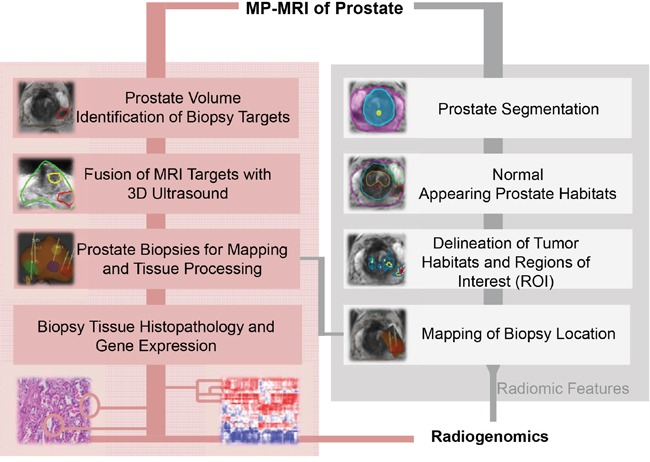
Experimental Design Multiparametric (mp)MRI, consisting of anatomical (T2-weighted), perfusion (Dynamic Contrast Enhanced [DCE]-MRI) and diffusion (Diffusion Weighted Imaging [DWI]) imaging sequences is acquired on 3T scanner. Upper left-hand side (shaded in pink) denotes the procedures for mpMRI-ultrasound fused targeted biopsies. The steps for radiomic analysis are presented at the right hand side in grey. Histopathology results, gene expression analysis and radiomic features are combined in the radiogenomic analysis.

**Figure 2:**
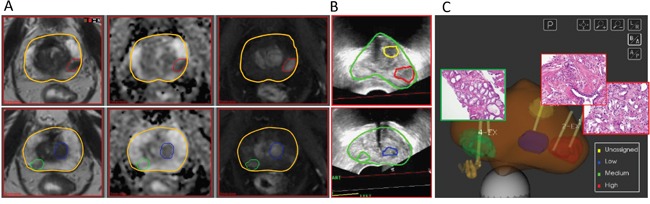
Delineation of biopsy targets on mpMRI and fusion of targets on 3D TRUS **A.** Screenshots from ProFuse software (Eigen, Grass Valley, CA) for fusion of mpMRI delineated prostate Regions of Interest (ROIs) for targeted biopsy. Two axial slices, going from base (top) to apex (bottom) are displayed. The prostate volume is outlined (yellow contour); (Left) T2-weighted MRI; (Center) Apparent Diffusion Coefficient (ADC) derived from Diffusion Weighted Imaging (DWI); and (Right) Early enhancing image from Dynamic Contrast Enhanced (DCE-)MRI. The volumes in red, green and blue are assigned high, medium and low probability for cancer; **B.** A screenshot from Artemis (Eigen, Grass Valley, CA), displaying the 3D TRUS views corresponding to the axial slices in (A) after non-rigid fusion of the prostate boundaries on MRI and ultrasound. The targets are transferred from mpMRI to real-time ultrasound biopsy system; **C.** Schematic representation of the prostate and target volumes. (Note that the display contains a ROI in yellow is with unassigned probability). Yellow lines indicate biopsy needle tracks (1 needle in green, 2 in red and 1 in blue); The corresponding N&E slides at 20 x magnification from green target (left; Gleason Score 6) and red targets (right, Gleason Score 7).

The radiogenomics workflow is schematically represented in Figure 1. An mpMRI exam of the prostate, consisting of anatomical (T2w), perfusion (DCE-MRI) and diffusion (DWI) was acquired.

(Figure 1, *pink shading*) Prostate and suspicious for cancer regions were outlined in ProFuse (Eigen, Grass Valley, CA), a multi-modality image fusion software. MRI and 3D-Ultrasound of the prostate were co-registered, using deformable fusion. Tissue from the identified targets was obtained for pathology and gene expression analysis. The procedure for selecting MRI-guided biopsy targets is outlined in Figure 2.

(Figure 1, *grey shading*) Radiomics data were extracted in MIM system (MIM Software, Inc., Cleveland, Ohio). Prostate, peripheral zone (PZ) and urethra were manually contoured on T2w. TZ volume was determined by subtracting the PZ and urethra from the prostate volume. In addition, volumes of Normal Appearing Tissue (NAT) in PZ and TZ were outlined. The tumor habitats are determined as described in *Methods*. Briefly, tumors were characterized by high vascular perfusion/permeability and rapid contrast washin and gradual washout on DCE-MRI, and then the suspicious areas are stratified as high, mid and low probability for cancer. Similarly, areas of restricted diffusion, associated with tumor growth and cancer cell proliferation were delineated on ADC maps and assigned high, medium and low probabilities based on established values (≤800, >800 to ≤1000, and 1000 to ≤1200 μm^2^/s). Tumor habitats were identified as the intersections of high perfusion and low diffusion areas and the corresponding probabilities were assigned. The biopsy regions of interest (ROIs) were derived from the identified habitats and other imaging information. The needle biopsy path was back-projected onto the MRI. Some of the radiomic features were from the biopsied tumor habitats and some were from normal appearing regions of the prostate.

### Patient samples

Between September 2012 and March 2014, 37 patients underwent mpMRI-guided prostate biopsies at the University of Miami and six patients met the following selection criteria: presence of at least three positive biopsies within a prostate with either *(i)* at least two distinct lesions found on imaging; or *(ii)* two distinct GS within a single lesion. Total of nineteen biopsies were identified and the summary of the patient characteristics is presented in Table 1. The Gleason Score (GS) from re-reviewed H&E slides prior to gene expression analysis shows that the selection criteria did not hold in some cases.

**Table 1:** Patient clinical characteristics

Patient ID	Age (years)	PSA (ng/ml)	T-category	Biopsy Location	GS^†^ (selection)	GS^‡^ (review)
P1	76	10.8	T1c	Left mid posterior PZ	GS 7	GS 7
				Left mid posterior PZ	GS 7	GS 7
				Right apex posterior PZ	GS 7	GS 6
P2	85	6.2	T1c	Left apex posterior PZ	GS 8	GS 8
				Left apex posterior PZ	GS 8	no tumor^#^
				Left mid posterior PZ	GS 8	no tumor^#^
P3	67	4.4	T1c	Left apex lateral PZ	GS 7	GS 7
				Left mid lateral PZ	GS 7	GS 8
				Left mid lateral PZ	GS 7	GS 7
P4	61	5.1	T1c	Right apex posterior PZ	GS 6	GS 6
				Right mid posterior PZ	GS 6	GS 6
				Left apex lateral PZ	GS 6	GS 6
				Left mid anterior TZ	GS 7	GS 6
P5	65	4.2	T1c	Left mid anterior TZ	GS 7	GS 6
				Left mid anterior TZ	GS 6	GS 6
				Left base anterior TZ	GS 6	GS 6
P6	72	10.8	cT2b	Left apex posterior PZ	GS 7	GS 9
				Left mid posterior PZ	GS 7	GS 7
				Left mid posterior PZ	GS 7	GS 9

*Abbreviations:* PSA = Prostate Specific Antigen; GS = Gleason Score; PZ = Peripheral Zone; TZ = Transition Zone

* Biopsies in different color are taken from distinct lesions in the prostate;

† GS at selection of patients for gene expression analysis;

‡ GS at re-review;

# Not enough tumor for gene expression analysis.

### Genomics features and association with gleason score

Out of 19 biopsy samples obtained, 17 samples yielded sufficient RNA (>100ng) for amplification and hybridization to Affymetrix Human Exon 1.0 ST microarrays (Table 1). All 17 biopsy samples passed microarray quality control metrics as described in *Methods*. Unsupervised clustering was performed in order to compare tumor expression patterns between patients and within patients. Hierarchical clustering using Pearson's correlation as the distance metric was performed using all genes and phylogenetic trees were created for visualization (Supplementary Figure S1A). The unsupervised clustering grouped samples on the individual branches of the tree (a measure of similarity) based on patient origin. In comparison, when clustering analysis was performed using only prostate cancer related genes, patient samples clustered based on Gleason Score (Supplementary Figure S1B). Finally, the 22 genomic expression feature Decipher^®^ (GenomeDx, San Diego, California) test clustered samples by Gleason score (Supplementary Figure S1C).

**Figure 3:**
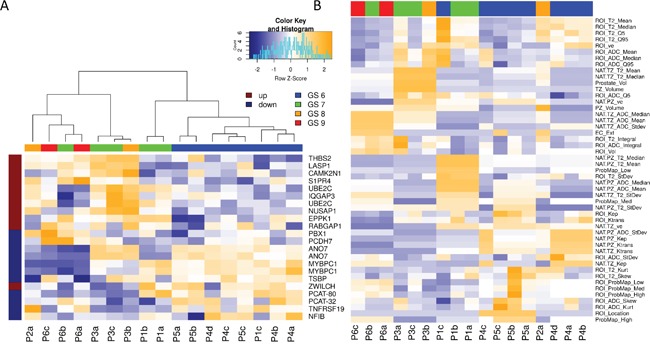
Hierarchical clustering of Genomic and Radiomic features and patient samples **A.** Hierarchical clustering on expression of the Decipher genes and patient samples. Note how biopsies are grouped by Gleason Score. Decipher genes, known to be highly expressed in more aggressive cancers (marked in dark red) are more highly expressed in higher GS samples and vise versa; **B.** Unsupervised clustering of all radiomic features revealed mostly clustering by patient.

Expression patterns of the 22 gene Decipher panel are illustrated as a heatmap in Figure 3A. Hierarchical clustering segregated the cohort into Gleason 6 and Gleason 8-9 disease. Gleason 7 samples segregated in both low and high risk clusters, in keeping with the genetic heterogeneity of this subtype. In addition to GS, the Decipher expression patterns also segregated by risk category, suggesting strong correlation between Gleason and Decipher score. Decipher and Gleason scores were also consistent with previous evaluations of tumor specimens from RP (Supplementary Figure S2) [13]. Low Decipher scores were significantly associated with Gleason scores (p-value = 8.23e-05) and all 8 samples with GS 6 were classified as Decipher low risk, based on previously reported cut-points for Decipher risk groups (Decipher score < 0.45). Decipher score was also significantly positively correlated with PSA (Spearman correlation p-value = 0.037, data not shown).

### Radiomic features and association with gleason score

Using propriety extensions in MIM, we created a radiomics pipeline for extraction of 49 quantitative imaging features (Table 2). Twenty five features were first used to characterize the prostate (*prostate level imaging features* - light gray, top half of Table). Within each prostate 24 imaging features were then used to characterize each region of interest (*biopsy ROI* – dark gray, bottom half of Table 2). Features belonged to one of the four general categories: *(i)* volumes; *(ii)* intensity; *(iii)* perfusion; and *(iv)* diffusion. Each of these parameters was used in combination to define specific nomenclature for each unique feature. (For example, NAT.PZ_ADC_Mean refers to the mean ADC value in the contour of naturally appearing tissue (NAT) in the PZ).

**Table 2:** Description of imaging features, extracted from various prostate regions utilizing mpMRI. Patient-specific features are shown in the upper part of the table; radiomic features of the targeted biopsy region are shown below

	Prostate Region(s)	Imaging Modality	Imaging Feature (units)	Number of features
**PATIENT** **(Total Features = 25)**	(1) Prostate (2) Peripheral Zone (3) Transition Zone	T2w	Volume (cc)	3
	(1) NAT-PZ (2) NAT-TZ	T2w ADC	Mean, StDev, Median	12
	(1) NAT-PZ (2) NAT-TZ	DCE-MRI	K^trans^(min^-1^) k_ep_(min^-1^) v_e_(%)	6
	***Probability Maps:*** (1) Low (2) Mid (3) High	DCE-MRI/ADC	Volume (cc)	3
	Extracapsular Extension	T2w	Yes *vs* No	1
**BIOPSY SPECIFIC ROI** **(Total Features = 24)**	ROI	T2w/Ultrasound	Volume (cc)	1
		T2w ADC	Mean, Median, StDev, Q5, Q95, Integral Skewness, Kurtosis	16
		DCE-MRI	K^trans^(min^-1^) k_ep_(min^-1^) v_e_(%)	3
	***ROI ∩ ProbMaps:*** (1) Low (2) Mid (3) High	DCE-MRI/ADC	Volume (cc)	3
	ROI location	T2w	PZ *vs* TZ	1

*Abbreviations:* ADC = Apparent Diffusion Coefficient; TZ = Transition Zone; DCE-MRI = Dynamic Contrast Enhanced MRI; NAT = Natural Appearing Tissue; mpMRI = multiparametric MRI; PZ = Peripheral Zone; ROI = Region of Interest.

Unsupervised clustering of radiomic features revealed clustering by patient (Figure 3B). When only ROI features were retained, the samples didn't show any clear clustering pattern.

### Association of radiomic features and commercially available prostate cancer classifiers

**Figure 4:**
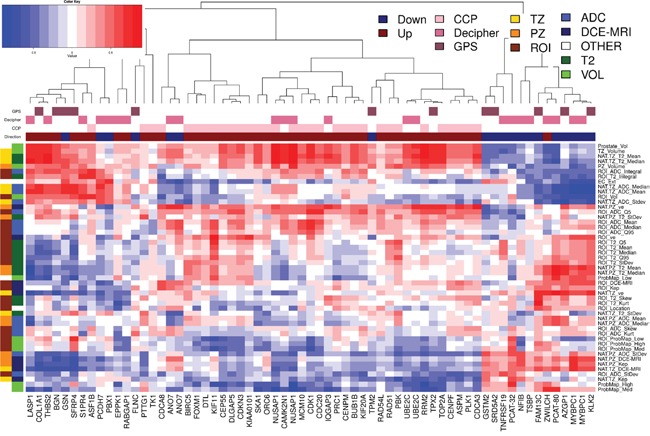
Pearson's correlation analysis of imaging features and 65 genes from commercially available prostate cancer classifiers Hierarchical clustering on Pearson's correlation distance between radiomic features and genes from commercially available prostate cancer classifiers: CCP (Cell Cycle Progression), Decipher and GPS (Genomic Prostate Score). Genes in these signatures that are up-expressed in aggressive cancers are indicated by a dark red box over the gene's column while those that are down-expressed are indicated with a blue box. Groups of radiomic features are indicated along the dendrogram on the left. Group1 (left) connects the radiomic feature with location (TZ, PZ and ROI); Group 2 is related to the image modality/function: T2w, ADC and DCE-MRI.

Genes from three commercially available prostate cancer prognostic signatures, Polaris Cell Cycle Progression (CCP), Decipher, and Genomic Prostate Score (GPS), were assessed for their relationship to the radiomic features. In Figure 4 we demonstrate significant associations between quantitative imaging features and genes associated with adverse outcome. The three clinically available signatures are prognostic for adverse prostate cancer outcomes and contain genes that are over-expressed in aggressive prostate cancers and genes that are under-expressed in aggressive prostate cancers. Figure 4 depicts correlations between these genes and quantitative imaging features. 445 of these correlations have a significant unadjusted p-value (p<0.05) and 64 correlations have a significant p-value after adjustment for multiple testing using False Discovery Rate (FDR) adjustment.

Genes in these signatures that are over-expressed in aggressive cancers are indicated by a dark red box over the gene's column while those that are down-expressed are indicated with a blue box. Groups of radiomic features are indicated along the dendrogram on the left: Group1 (left) connects the radiomic feature with location (TZ, PZ and ROI) and Group 2 is related to the image modality: T2w, ADC and DCE-MRI. The primary cluster of the genes segregates them into genes over-expressed in more aggressive cancers (left cluster) and under-expressed in aggressive cancers (left cluster). The over-expressed cluster is further subdivided into two clusters, where the right side is enriched for CCP genes (28/31 genes). As expected the CCP genes are highly positively correlated to each other and hence have similar relationships with the radiomic features. Both Decipher and GPS contain genes that represent the expression patterns found in each of these three main clusters, capturing signal from the under-expressed genes, CCP, and other over-expressed genes.

The radiomic features in Figure 4 clusters into 2 main groups. The top group is enriched for TZ and volumetric features which are mainly positively correlated to the prostate cancer genes over-expressed in more aggressive cancers. The bottom cluster contains mainly PZ and ROI features. Both the top and bottom clusters are further subdivided into radiomic features which are positively and negatively associated with cell cycle progression genes.

### Association of radiomic and genomic data

**Figure 5:**
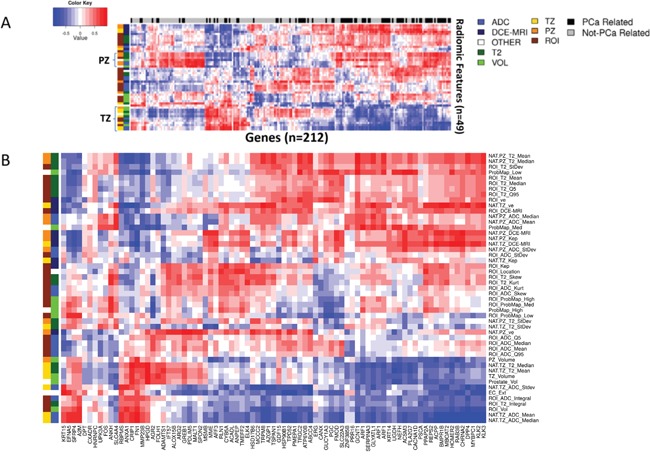
Pearson's correlation analysis identified genomic features that were highly associated with the imaging features **A.** Hierarchical clustering on Pearson's correlation distance between radiomic features and genes with significant expression values. Prostate cancer related genes are indicated in black along top dendrogram. Two clusters with reciprocal behavior are related mostly to PZ and TZ radiomic features; **B.** Prostate cancer gene enriched region of the heatmap in (A). Groups of radiomic features are indicated along the dendrogram on the left. Group1 (left) connects the radiomic feature with location (TZ, PZ and ROI); Group 2 is related to the image modality: T2w, ADC and DCE-MRI.

The relationship between genomic and radiomic features was further investigated by summarizing the 1.4M probesets to 22,011 annotated genes, using Affymetrix core level summaries, and filtering for significant expression by removing any genes with median expression <0.25 and Interquartile Range (IQR) <0.5. Pearson's correlation distances were applied between the remaining 212 genomic and 49 radiomic features. Two-way hierarchical clustering of these distances is illustrated as a heatmap in Figure 5A. Interestingly, 79/212 (37%) of these genes, marked with black bars along the top of the heatmap, were prostate cancer related and tended to cluster together. The two clusters, marked with TZ and PZ, display a degree of reciprocal trends: of the 212 selected genes, 121 had a significant positive correlation to radiomic features related to the PZ and a significant negative correlation to radiomic features related to the TZ (Supplementary Table S1). A two-way hierarchical clustering of correlation distances between radiomic features and only those genes that are prostate cancer related (annotated as black bars at the top in Figure 5A) is depicted in Figure 5B. Notably, one group of genes with very strong negative correlations (≤-0.9, Supplementary Table S2) to radiomic features were found to be associated with the AR signaling genes: KLK2, KLK3, HOMER2, BMPR1B, or belong to validated prostate cancer classifiers: [4, 14] CHRNA2, MT1H, DPP4, MYBPC1. Genes with very high positive correlations (≥ 0.9) to radiomic features were: TRPM8, DPP4, GCNT1.

**Figure 6:**
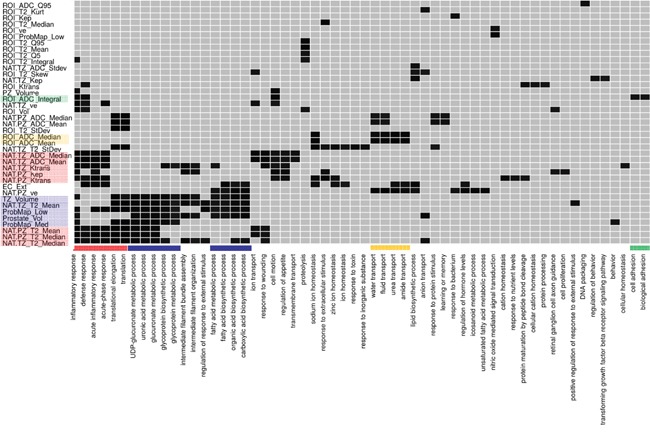
Enrichment analysis using Database for Annotation, Visualization and Integrated Discovery (DAVID) identified enrichment of radiomic features for different Gene Ontology (GO) biological processes Enrichment analysis was performed for each radiomic feature using the list of significantly expressed genes with a significant correlation to that radiomic feature. Significantly enriched (p-value < 0.05) biological processes are shown in black. radiomic features with no enriched processes are not shown. The association between radiomic features and biological processes are denoted with the same color. For example, for PZ and TZ radiomic features (highlighted in red), biological processes such as immune/inflammatory and cell-stress responses were significantly enriched GO terms (red bar).

Gene ontology (GO) analysis further identified distinct GO biological processes, associated with prostate radiomic features (Figure 6). [11, 15] PZ and TZ radiomic features (highlighted in red), biological processes such as immune/inflammatory and cell-stress responses were significantly enriched GO terms (red bar). This relationship suggests a potential field effect related to tumor-induced stress responses in the prostate. A series of metabolic and biosynthetic processes, indicated by the blue bars, were associated mainly with volumetric radiomic features, including those of the tumor habitats (probability maps volumes). Again, these processes could be activated in response to the growing tumor and its modulating effect on its microenvironment. Lastly, several fluid transport (yellow bar) and adhesion (green bar) processes are associated with the ROI radiomic ADC features.

## DISCUSSION

Prostate cancer is often multifocal and heterogeneous, and thus presents a challenge in identifying regions for biopsy that are most likely to be determinate of outcome. Herein, we describe a two-step process to identify regions of prostate cancer most likely to harbor aggressive disease and, thus, both mitigate the impact underdiagnosis and reduce the personal and health system costs of unnecessary prostate biopsies.

Standard transrectal ultrasound (TRUS) guided prostate biopsies are imprecise with 30% or more of prostate tumors sampled being isoechoic [16] and a roughly 50:50 chance of documenting cancer in hypoechoic lesions [17]. Since the needle biopsy cannot be directed reliably to a tumor focus, a template systematic biopsy of the gland is now routinely used, but even in these settings, the highest grade and/or volume lesions are often missed. Furthermore, high grade and high volume index lesions, even when identified may not be determinate of outcome [18], suggesting that alternative strategies are needed. Most recently, mpMRI has emerged as the best modality to localize cancer within the prostate, allowing us to sample directly from regions of the prostate that are most likely to harbor an aggressive tumor. There is still concern for tumor heterogeneity within a specific MRI target, and identifying sub-targets within a region of suspicion on the MRI remains a challenge.

While advances have been made in structuring the assessment and biopsy of abnormalities in the prostate using mpMRI via PI-RADS [19], there remains considerable subjectivity and no incorporation of quantitative information. We hypothesized that quantitative imaging characteristics may be applied to better characterize imaging phenotypes or habitats in the prostate. We linked radiomic features to existing genomic and clinical information to improve risk stratification, as has been previously described by us [10] and others for different tumor types [19].

Prostate “habitats” were identified using images, acquired with multiple acquisition parameters assuming that distinct combinations of these quantitative parameters represent different physiologies. This approach has successfully improved outcome prediction in glioblastoma [20] and sarcoma [21]. In our implementation, habitats suspicious for malignancy were delineated based on reduced diffusion and increased perfusion. While intra and inter-patient reproducibility of DCE-MRI is of concern, a partial mitigating factor for DCE-MRI use is that all patients in the study were scanned on the same magnet with the same sequences and analyzed with the same algorithm. T2w was not included in the procedure as T2w information is not strictly orthogonal to DCE-MRI and DWI information.

The analysis of the Pearson correlations between genomic and radiomic features confirmed the presence of strong prostate cancer signal in the radiomic features. The significant correlations between quantitative imaging features and the genes in the three clinically available signatures associated with adverse outcome confirm the relevance of the radiomics features to cancer aggressiveness. Interestingly, the Gene Ontology (GO) analysis revealed that from all radiomics features of the biopsy region, the ADC values were most highly associated with distinct biological processes. With increase of the sample size and the robustness of the analysis, the developed workflow can be utilized to identify features with the highest added value for associations with gene expression and gene ontology.

For radiomic analysis, the normal appearing regions in the prostate are also considered. The rationale for this novel use of imaging data, is that the tumor microenvironment selects for cancers with distinct phenotypes and that physiologically distinct regions observed in images reflect underlying pathophysiologies. For the prostate, stromal epithelial interactions have been shown to contribute to tumor behavior, [22] demonstrating the importance of the microenvironment. On the other hand, the phenomenon of field cancerization has been observed in prostate cancer on both genomic and proteomic levels [23]. To the best of our knowledge, this is the first radiomic study that includes extraction of imaging features from Normal Appearing Tissue (NAT). Gene Ontology (GO) analysis revealed that series of GO biological processes are distinctly associated with PZ and TZ radiomic features, suggesting that NAT regions may contribute to tumor phenotype or vice versa. These results are highly intriguing and warrant further investigation in larger studies. While some of the covariates on patient level are linked and raise concerns about oversampling, in this exploratory analysis we aimed to create a pipeline for comprehensive analysis of the imaging data of the prostate. As such, we extracted and retained imaging characteristics potentially important for the tumor environment. With an increased number of patients and biopsy samples, the concern about oversampling will be diminished.

In conclusion, quantitative features derived from mpMRI guided biopsies are associated with established clinical-pathologic characteristics (e.g., Gleason score). Further, radiomic features were correlated with known prognostic gene expression patterns in prostate cancer. The novelty of this application is threefold: *(i)* integration of radiomic features from multiple MRI sequences; *(ii)* extraction of radiomic features from 3D volumes (rather than from a single image slice); and *(iii)* identification of radiomic features in normal appearing tissues that are associated with high risk gene expression profiles. While encouraging, validation of this approach in a larger dataset is required to demonstrate significant improvement over existing clinic-pathological and genomic risk stratification.

## MATERIALS AND METHODS

### Patients

This retrospective study was HIPAA compliant and approved by the institutional review board with a waiver of written informed consent.

### Multiparametric MRI of the prostate

T2w MRI provides an excellent depiction of prostate anatomy with lower signal intensity in prostate cancer. [24] Diffusion Weighted Imaging (DWI) is sensitive to water molecule diffusion and the derived Apparent Diffusion Coefficient (ADC) values are significantly lower in tumor than in normal prostate due to restricted water diffusion. The lower the ADC value, the greater the chance of diagnosing Gleason score (GS) 7 disease [25-27]. Dynamic contrast enhanced (DCE)-MRI has also been applied to discriminate normal from malignant prostate tissues, with earlier and greater enhancement followed by washout seen in the latter. DCE-MRI measures vascularity and hence angiogenesis. Both DWI and DCE have a relatively high sensitivity and specificity for prostate cancer [25, 28-30]. mpMRI that includes T2-weighted, T1 non-contrast, DCE-MRI, and DWI sequences results in higher sensitivity, specificity and accuracy of tumor localization [30, 31].

mpMRI of the prostate was performed on a Discovery MR750 3.0T MR scanner (GE Medical Systems, Milwaukee, WI, USA) with 32-channel phased array pelvis coil. A typical exam consisted of:

- Axial T2w-MRI of the male pelvis: resolution 1.25×1.25×2.5 mm; Field of View: 320×320 mm; slice thickness - 2.5 mm (no gap); 72 slices; repetition time (TR) 10800 ms/echo time (TE) 83 ms; flip angle 120°;
- Axial T1w gradient echo MR images are acquired with identical spatial resolution, spacing and image size as the T2w MR images. Sequence parameters are: TR/TE 4.1/2.8 ms; flip angle 12°;
- DWI - Single-shot echo-planar imaging is performed utilizing the diffusion-module and fat-suppression pulses. Water diffusion in four directions is measured by using *b* values of 50, 500, and 1000 s/mm^2^, TR/TE 9500/53, a parallel imaging factor of two, 36 sections, 2.5-mm-thick sections, and an in-plane resolution of 2.5×2.5 mm. ADC maps were automatically calculated utilizing software onboard the GE MRI console with use of all three *b* values;
- DCE-MRI - Prior to contrast material injection, one set of T1 MRI are acquired as pre-contrast image set. The rest of the data are acquired following intravenous bolus injection of a paramagnetic gadolinium chelate - 0.1 mmol of gadobenate-glumine (Bracco Diagnostics Inc., Princeton, New Jersey) per kilogram of body weight. The contrast is administered with a power injector (Spectris, Medrad Inc., Warrendale, Pennsylvania) at 2 mL/s and followed by a 20-mL saline flush. Eleven to twelve post-contrast imaging datasets are collected.

### Delineation of regions of interest on mpMRI for biopsy targets

Regions of Interest (ROIs) based on established mpMRI analysis criteria and informed by software showing habitats suspicious for harboring tumor were contoured in ProFuse (Eigen, Sun Valley, CA) multi-modality image fusion software. The procedure is illustrated in Figure 2A. The prostate volume is outlined (yellow).

### mpMRI-ultrasound fused targeted biopsy

MRI/Ultrasound image fusion in Artemis system (Eigen, CA) is part of the planning process during the biopsy procedure [32]. A 3D transrectal ultrasound (TRUS) is acquired just prior to biopsy by reconstructing sweeps of 2D to 3D. The prostate volume is semiautomatically segmentated [33] on TRUS and both these volumes are fused after specification of four or more corresponding points along the gland boundary. The triangulated gland surfaces from both modalities are registered using an adaptive focus deformable model [34]. During biopsy, as the operator visualizes the real time ultrasound volume on screen, motion correction runs automatically every few hundred milliseconds to compensate for any movement of the prostate after the acquisition of the 3-D volume. Finally, the original 3-D TRUS volume and warped MRI volume are both readjusted to correspond with the real time 2D ultrasound image. Figure 2B shows the results of co-registration, where MRI targets are visualized on TRUS. The lesion is targeted using ultrasound monitoring to ensure the correct depth and course of the needle. The system computes the needle trajectory, its core position and depth with a high degree of accuracy (Figure 2C). Importantly, the XYZ coordinates of the biopsy site are recorded in 3D for future reference, treatment planning and monitoring.

### Prostate habitats

The concept and practice of defining specific ‘habitats’ from radiological images was relatively recently introduced and we used the approach to facilitate applying radiomics to prostate cancer analysis [10]. This approach requires the combination of co-registered images from multiple modalities, with each one contributing a piece of orthogonal information. For this reason, MRI is a technique of choice because multiple pieces of co-registered orthogonal data can be generated in a single exam. For example, DCE-MRI identifies regional distributions of blood flow, and lack of blood flow. ADC, measured via diffusion MRI, is a powerful method to interpolate the density of diffusion barriers (i.e. cells) and hence provides information that may be biologically, but not physically, related to DCE-MRI. T2 is sensitive to microsocopic perturbations in the magnetic field; this is affected by blood flow and cell density, but in a non-linear fashion. Hence, T2 information is not strictly orthogonal to DCE-MRI and ADC and in the current implementation reduced diffusion and increased perfusion are considered for delineation of habitats suspicious for malignancy. The habitats concept was initially introduced to map the tumor heterogeneity [35]. Tumors can be described as complete ecosystems, containing cancer cells, stromal cells, vasculature, structural proteins, signaling proteins and physical factors such as pH and oxygen [36]. In this work, we extend the use of habitat's concept to the entire prostate as an important component of tumor environment, including ‘normal’ appearing tissues in the PZ and TZ.

First, the prostate, PZ and urethra were manually contoured in 3D in MIM software (Supplementary Figure S3A). Contrast-vs-time curves were extracted from all pixels in the prostate volume and unsupervised pattern recognition approach decomposes the data as a product of several temporal patterns and their relative contribution, amplitude in each pixel [37]. Let A be the amplitude of the ‘tumor’ pattern, which is associated with the well perfused temporal pattern (rapid wash in and gradual wash out of the contrast). Thresholds for areas of high, mid and low risk for aggressive tumor are estimated as mean(A)+k*stdev(A), where k=2, 1.5 and 1. The tresholded maps of A are overlaid in pink (high risk); green-yellow (mid) and blue (low) on the earliest enhancing image in the DCE-MRI (Supplementary Figure S3B). Similarly, the ADC map was thresholded at 800, 1000 and 1200 μm^2^/s based on literature and empirical observations in our group [38-43]. The tresholded maps are overlaid on the ADC map in pink (high risk); green-yellow (mid) and blue (low) (Supplementary Figure S3C). Finally, the areas of intersections between perfusion and diffusion were considered the volumes of high, mid and low probability for high risk cancer (Supplementary Figure S3D). ROI, NAT_PZ and NAT_TZ are presented in red, green and orange in Supplementary Figure S3E.

### Extraction of radiomic features

The summary of radiomic features is presented in Table 2. A total of 49 features were computed for these volumes of interest. The volumes of prostate, PZ, TZ and ROI were estimated in MIM (n=4) (Supplementary Figure S3A, S3D). Mean, median and stdev of the intensities of T2w and ADC in NAT_PZ, NAT_TZ and ROI were calculated (n=18). In addition for the ROI, the top and bottom 5 percentile (Q5 and Q95), skewness, kurtosis and integral were recorded for both T2w and ADC (n=10). The ‘extended Tofts model’ [44, 45] was applied to the averaged DCE-MRI curves within NAT_PZ, NAT_TZ and ROI. Using synthetic Parker fixed population average Arterial Input Function (AIF) [46] we have shown that a valid compartmental modeling can be carried out even at the lower temporal resolution of the data [47]. Three features from the pharmacokinetic analysis (K^trans^ – Volume transfer constant between plasma and Extracellular Extravascular Space (EES), k_ep_ – Rate constant between EES and plasma and v_e_ – the fraction of the EEC) were estimated for NAT_PZ, NAT_TZ and ROI (n=9). The volumes of the probability maps and their intersections with ROI were also included (n = 6). Two semantic features were also included in the analysis: extra capsular extension and location of the lesion (n=2). Further we investigated the radiomics features for redundancy. In Supplementary Figure S4 the auto-correlation matrix of the 49 features is presented as a heat-map. Mean and medians of intensities were highly correlated as well as volumes of the prostate, peripheral zone and transition zone. Overall, there were less than 4% of pairs (46/1176 comparisons) of significantly correlated imaging features (p-value (Holm's p-value adjustment) cutoff of 0.05). As the goal of this exploratory analysis was to create a pipeline for comprehensive analysis of the imaging data of the prostate and in view of the small number of redundant pairs, we retained all radiomics features for subsequent analysis.

### RNA extraction and microarray hybridization

From the original study (n = 19), RNA was available for microarray from 17 biopsies (6 unique patients). As previously described [4, 48], after histopathological re-review by an expert genitourinary pathologist, tumor was macrodissected from surrounding stroma from 3–4 10 μm tissue sections from a region with maximum tumor content for total RNA extraction.

RNA extraction and microarray hybridization was performed using clinical-grade techniques in a Clinical Laboratory Improvement Amendments (CLIA)-certified laboratory facility (GenomeDx Biosciences, San Diego, CA, USA). CLIA certification was obtained through the Centers for Medicare and Medicaid Services through standard procedures, and laboratory facilities satisfied all criteria required for certification. Total RNA was subjected to amplification using the WT-Ovation Formalin Fixed Paraffin Embedded (FFPE) v2 kit together with the Exon Module (NuGen, San Carlos, CA) according to the manufacturer's recommendations with minor modifications. Amplified products were fragmented and labeled using the Encore Biotin Module (NuGen, San Carlos, CA) and hybridized to Human Exon 1.0 ST GeneChips (Affymetrix, Santa Clara, CA) following manufacturer's recommendations. Human Exon GeneChips profile coding and non-coding regions of the transcriptome using approximately 1.4 million probe selection regions (PSRs), hereinafter referred to as features. All of the samples with available tissue and RNA, passed initial quality control. Quality control for microarray data was performed with Affymetrix Power Tools packages [49] and with internally developed metrics including percent present- the percentage of probes detected above the limit of detection [50]. The positive versus negative area under the curve (AUC) was used as an additional metric to assess microarray quality by measuring the signal between positive control probes, which measure the expression of housekeeping genes, and negative control probes, which measure anti-genomic sequences and hence should exhibit background intensity levels. The percent present observed in these biopsy samples was higher than the typical range seen in FFPE radical prostatectomy samples. The Human Exon 1.0 ST array data have been deposited in the Gene Expression Omnibus database under accession number GSE80683.

### Microarray normalization, removal of unreliable features and batch effect correction

Feature summarization and normalization were performed using the Single Channel Array Normalization (SCAN) algorithm, which normalizes each sample individually by modeling and removing probe- and array-specific background noise [51]. To calculate gene expression, we used Affymetrix Core level summaries for annotated genes. In order to remove known batch effects in the microarray data, the ComBat method from the sva package (sva_3.8.0) was used [52]. Features were further filtered for significant expression by removing any features with median expression < 0.25 and Interquartile Range (IQR) < 0.5.

### Radiogenomic analysis

In order to uncover the relationship between genomic features and radiomic features, Pearson's correlation distances between radiomic features and genomic features with significant expression were computed. The number of significant positive and negative correlations was determined using an FDR adjusted p-value cut-off of 0.05. To assess the correlation between radiomic features and existing clinical and genomic risk stratification biomarkers the features from three clinically available signatures were used. For visualization of the results, heatmaps were generated using two-way hierarchical clustering on the distances.

Sample clustering analysis was performed to evaluate clustering patterns of samples and patients. Hierarchical clustering was performed on Pearson correlation distances of all significantly expressed genes.

Gene Ontology enrichment analysis was performed using The Database for Annotation, Visualization and Integrated Discovery (DAVID) v6.7 along with RDAVIDWebServices R package (RDAVIDWebService_1.6.0) to identify biological processes that are associated with radiomic features. Genes that were significantly correlated (p-value < 0.05) with each radiomic feature were analyzed in DAVID and biological processes that were significantly enriched (p-value < 0.05) for each radiomic feature were identified.

## SUPPLEMENTARY FIGURES AND TABLES






