# Supplementary material for: Association of multiparametric MRI quantitative imaging features with prostate cancer gene expression in MRI-targeted prostate biopsies
Source: Oncotarget. 2016 Jul 11;7(33):53362–76. doi: 10.18632/oncotarget.10523 (PMC5288193; doi:10.18632/oncotarget.10523)
Supplement: Supplementary file 1 [file oncotarget-07-53362-s001.pdf]

# Association of multiparametric MRI quantitative imaging features with prostate cancer gene expression in MRI-targeted prostate biopsies

## SUPPLEMENTARY FIGURES AND TABLES

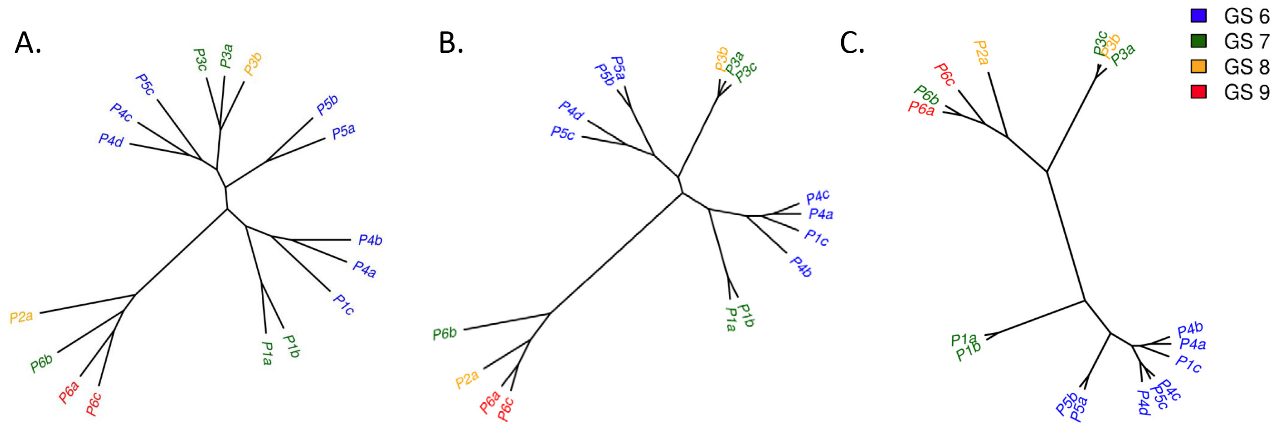


**Supplementary Figure S1: Clustering of biopsy samples. A.** Unbiased genomic cluster analysis with all genes groups the samples mainly by patient; **B.** When only prostate related genes are used, the samples tend to cluster and by Gleason Score (GS); and **C.** Decipher genes separate the samples by tumor aggressiveness.

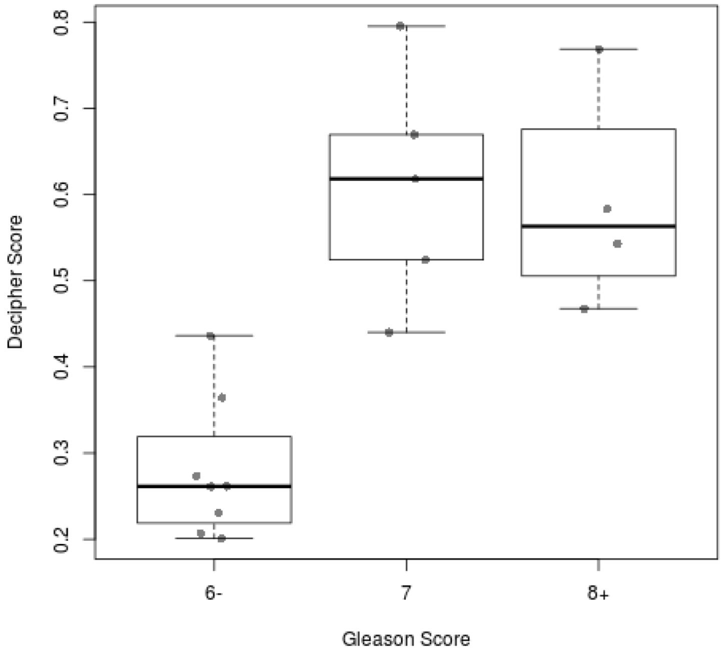


**Supplementary Figure S2: Association of Decipher score with Gleason score.** Low Decipher scores are significantly associated with lower Gleason scores. All 8 samples with GS 6 were classified as Decipher low risk (Decipher score < 0.45). The difference in Decipher scores between GS 6 vs. GS7+ was significant with p-value = 8.32e-05.

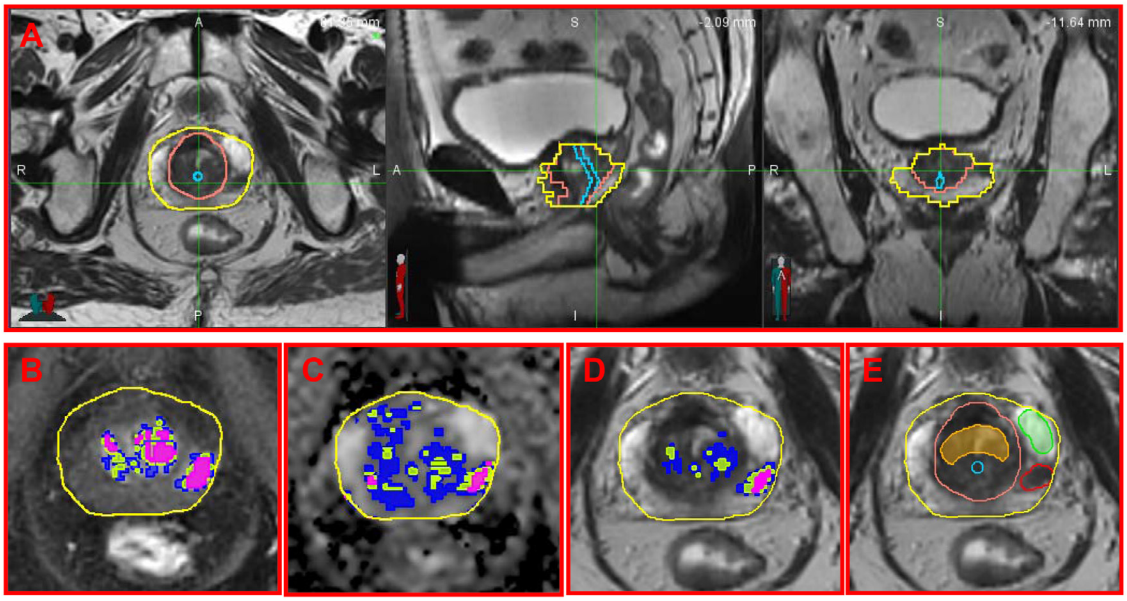


**Supplementary Figure S3: Delineation of prostate habitats. A.** Prostate (yellow), Peripheral Zone (PZ) (pink) and urethra (blue) were manually contoured in MIM software in three dimensions. The volumes are presented in three views: axial, sagittal and coronal. Prostate Tranzition Zone (TZ) is determined by subtracting PZ and Urethra from the prostate volume; **B.** Perfusion: Areas of high perfusion are presented on three levels (see *Methods*) for risk for tumor aggressiveness: high – pink, mid – green-yellow and low – blue, and overlaid on the early enhancing series form the DCE-MRI; **C.** Diffusion: Areas of low diffusion are presented on three levels (see *Methods*) for risk for tumor aggressiveness: high – pink, mid – green-yellow and low – blue, and overlaid on the ADC map. **D.** Volumes of high (pink), mid (green) and blue (low) probabilities for aggressive cancers are determined as intersections of the corresponding areas of high perfusion/ low diffusion; **E.** Biopsy target (ROI), normal appearing tissue - peripheral zone (NAT_PZ) and central region (NAT_TZ) are presented in red, green, and orange.

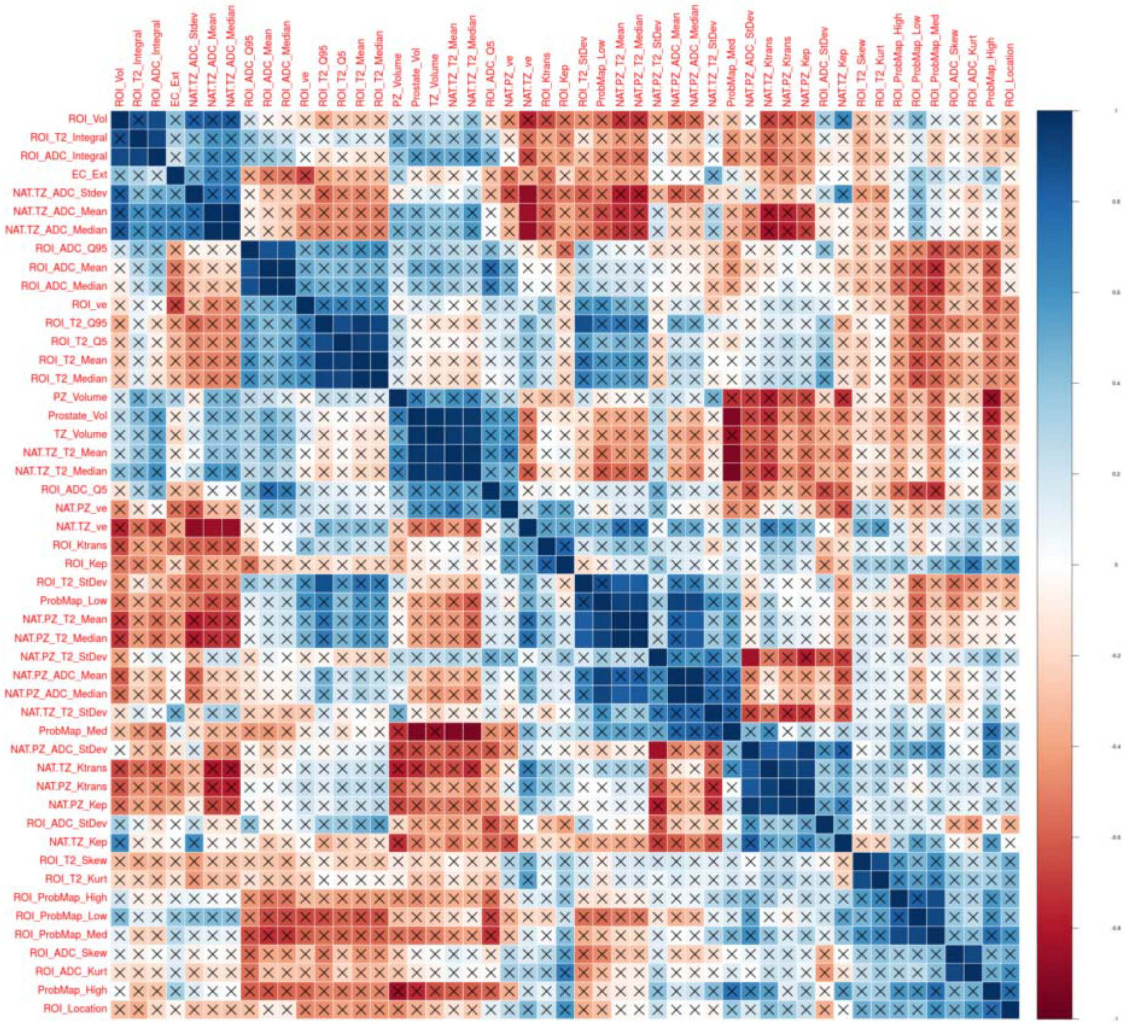


**Supplementary Figure S4: Heatmap of auto-correlation matrix of radiomic features.** Positive and negative correlations are shown in blue and red, respectively. Significantly correlated imaging features (p-value (Holm's p-value adjustment) cutoff of 0.05) are not marked with 'X'.

**Supplementary Table S1: Pearson's correlation between radiomic and genomic features**

See Supplementary Table 1

**Supplementary Table S2: Radiomic features, strongly correlated with expression of prostate cancer related genes**

See Supplementary Table 2
